# Palmitoylethanolamide reduces granuloma-induced hyperalgesia by modulation of mast cell activation in rats

**DOI:** 10.1186/1744-8069-7-3

**Published:** 2011-01-10

**Authors:** Daniele De Filippis, Livio Luongo, Mariateresa Cipriano, Enza Palazzo, Maria Pia Cinelli, Vito de Novellis, Sabatino Maione, Teresa Iuvone

**Affiliations:** 1 Department of Experimental Pharmacology, Faculty of Pharmacy, University of Naples Federico II, Via D. Montesano 49, Naples, Italy; 2 Department of Experimental Medicine, Section of Pharmacology, The Second University of Naples, via Costantinopoli 16, 80138 Naples, Italy; 3 Department of Biomorphological and Functional Sciences, Via Pansini University of Naples "Federico II", Naples, Italy; 4 Endocannabinoid Research Group, Via Campi Flegrei 34, 80078 Pozzuoli (NA), Italy

## Abstract

The aim of this study was to obtain evidences of a possible analgesic role for palmitoylethanolamide (PEA) in chronic granulomatous inflammation sustained by mast cell (MC) activation in rats at 96 hours. PEA (200-400-800 μg/mL), locally administered at time 0, reduced in a concentration-dependent manner the expression and release of NGF in comparison with saline-treated controls. PEA prevented nerve formation and sprouting, as shown by histological analysis, reduced mechanical allodynia, evaluated by Von Frey filaments, and inhibited dorsal root ganglia activation. These results were supported by the evidence that MCs in granuloma were mainly degranulated and closely localized near nerve fibres and PEA significantly reduced MC degranulation and nerves fibre formation. These findings are the first evidence that PEA, by the modulation of MC activation, controls pain perception in an animal model of chronic inflammation, suggesting its potential use for the treatment of all those painful conditions in which MC activation is an initial key step.

## Introduction

Mast cells (MCs) are well recognized as a "body guard" in host defense reactions, as inducers of innate and acquired immunity and tissue remodelling, because of their pivotal role in initiating allergic reactions. Moreover, in recent years, MCs have also been acknowledged to modulate the inflammatory process [1]. Their contribution to neuro-immune processes remains, however, less clear. There are many independent lines of evidence that indicate massive, bidirectional cross-talk between MCs and sensory nerves (SNs) suggesting that MCs and SNs may be functionally [2] and anatomically assembled within certain tissues [3,4]. In the skin, for instance, MCs are frequently co-localized with nerve fibres expressing substance P (SP) and calcitonin gene-related peptide (CGRP) and/or other peptidergic mediators [5]; moreover, activated MCs produce and release histamine, serotonin, and tryptase leading to SNs activation thus contributing to neurogenic inflammatory reactions [6]. Above all, MCs by releasing NGF and TNF-α, are thought to regulate SNs development, degeneration, and regeneration [7,8]. Therefore, MCs and SNs have been suggested to co-orchestrate a variety of physiological and pathological processes, such as hair follicle cycling, wound healing, stress responses and to contribute to the pathogenesis of inflammatory and autoimmune diseases [9,10].

In several animal models of inflammatory pain, including complete Freund's adjuvant-induced arthritis [11], carrageenin-induced paw oedema [12] and a rat model of cystitis [13,14], NGF expression was found to be increased. Moreover, it has been shown that over-expression of NGF results in a marked nerve fibre hyperplasia in the urinary bladder submucosa [15]. Recently, the administration of a new molecule sequestrating endogenous NGF has shown to reduce acute and chronic inflammatory processes and associated pain [16]. These studies suggest that peripherally produced NGF is involved in the development and maintenance of nociceptive sensory neuron sensitivity and that an up-regulation of NGF is responsible for alterations in pain-related behaviour [17]. Therefore, blockade of NGF production and/or its action has been proposed as a novel strategy to avoid nerve hypersensitivity induced by inflammation, and possibly as a novel non-canonical anti-inflammatory analgesic drug [18].

A class of molecules potentially able to control NGF synthesis and release is represented by ALIA compounds (from the acronym Autacoid Local Injury Antagonist), naturally-occurring lipid amides deriving from membrane fatty acids and structurally related to endocannabinoids. Palmitoylethanolamide (PEA) is considered the most important of the ALIAmides because of its ability to negatively modulate MC activation [19,20].

Although considerably more abundant than the endocannabinoid anandamide (arachidonoyl ethanol amide: AEA) or 2-arachidonoylglycerol (2-AG) in many tissues, the effects of PEA are less well know than those of AEA or 2-AG, probably due to its puzzling mechanism of action. In fact, PEA, although structurally related to AEA is inactive at the cannabinoid CB1/CB2 receptor site, but it exhibits several important pharmacological effects shared with endocannabinoid compounds, such as marked anti-inflammatory, anti-oedema and analgesic properties in a wide range of experimental models of inflammation [21]. There are several pieces of evidence indicating that PEA might represent part of a "parallel" endocannabinoid signalling system, with its own putative receptors.

To date, peroxisome proliferator-activated receptor-α (PPAR-α) and G protein-coupled receptor 55 (GPR55) have been suggested as endogenous putative receptors for PEA, since their stimulation contributes to some of PEA-induced anti-inflammatory and analgesic effects.

Moreover, it has been demonstrated that PEA significantly reduces peripheral pain through a mechanism that is enhanced by AEA and blocked by CB2 receptor antagonists [16]. The mechanism through which PEA exerts its anti-nociceptive effects remains unclear since it does not interact with the CB2 receptor. Therefore, it has been postulated that this compound might evoke analgesia through a still uncharacterized CB2-like receptor [16], probably expressed specifically on MCs [19]. Alternatively, several important actions of PEA seem to be mediated by none of these receptors, but independently of any receptor activation [22].

Therefore, the aim of our study was to investigate the anti-inflammatory and/or anti-allodynic properties of PEA in a model of chronic inflammation actively sustained by MC involvement, such as subcutaneous granuloma formation induced by λ-carrageenin in rats. Moreover, we evaluated the capability of PEA in reducing the granuloma-induced enhancement of pro-inflammatory mediators in neural and satellite cells of dorsal root ganglia (DRG) innervating granulomatous areas.

## Materials and methods

### Animals

Male Wistar rats (Harlan, Italy), weighing 200-220 g, were used in all experiments. Animals were housed three per cage under controlled illumination (12 h light/12 h dark cycle; light on 06:00 h) and standard environmental conditions (ambient temperature 20-22°C, humidity 55-60%) for at least 1 week before the beginning of the experiments. Chow and tap water were available ad libitum. Animal care as well as all experiments were in accordance with European Community Council Directive 86/609/EEC and efforts were made to minimize animal suffering and to reduce the number of animals used.

### Sponge implantation surgery

Sponges were implanted as previously described by De Filippis et al. [23]. Briefly, two polyether sponges (0.5 × 1.5 × 2.0 cm and 0.035 ± 0.002 g), previously sterilized by autoclaving for 20 min at 120°C, were subcutaneously implanted on the back of rats (*n *= 12-18 for group) under pentobarbital anesthesia (60 mg/kg). A volume of 0.5 mL/sponge of vehicle (saline), λ-carrageenin (Sigma-Aldrich, St Louis, MO, USA) 1% w/v in pyrogen-free saline with or without 100 μl of PEA solution (200, 400 and 800 μg/ml) was injected into each sponge. Rats were sacrificed in an atmosphere of CO_2 _96 h after the sponge implant. The tissue around the sponge showing granuloma was dissected out, weighted, quickly frozen in liquid nitrogen, and stored at -80°C.

### Histological investigation

The granulomatous tissue around the sponge was removed and fixed in 10% formalin. Paraffin-wax sections of thickness 4-6 μm were cut and stained with toluidine blue for the evaluation of MCs and with hematoxil and eosin to study new nerve formation.

### Western blot analysis

Granulomatous tissues were weighed and rapidly homogenized in 60 μl ice-cold hypotonic lysis buffer [10 mM HEPES, 1.5 mM MgCl_2_, 10 mM KCl, 0.5 mM phenylmethylsulfonyl fluoride, 1.5 μg/mL soybean trypsin inhibitor, pepstatin A 7 μg/mL, leupeptin 5 μg/mL, 0.1 mM benzamidine, and 0.5 mM dithiothreitol (DTT)] and incubated on ice for 45 min. After this, the cytoplasmic fractions were obtained by centrifugation at 13000 g for 1 min and the protein concentration in the samples was determined with a Bio-Rad assay kit according to the manufacturer's instructions. Immunoblotting analysis of NGF, PGP 9.5 and tubulin were performed on a cytosolic fraction of the cultured specimens. Cytosolic fraction proteins were mixed with gel loading buffer (50 mM Tris, 10% SDS, 10% glycerol 2-mercaptoethanol, 2 mg/mL bromophenol) in a ratio of 1:1 v/v, boiled for 5 min and centrifuged at 10000 g for 10 min. The protein concentration was determined and equivalent amounts (50 μg) of each sample were separated under reducing conditions on 12% SDS-polyacrylamide minigel. The proteins were transferred onto a nitrocellulose membrane according to the manufacturer's instructions (Bio-Rad Laboratories, Hercules, CA, USA). Depending on the experiments, the membranes were blocked by incubation at 4°C overnight in high salt buffer (50 mM Trizma base, 500 mM NaCl, 0.05% Tween-20) containing 5% bovine serum albumin; they were then incubated for 1 h with anti-NGF (1:100 v/v) (Oncogene, San Diego, CA), anti-PGP 9.5 (1:2500 v/v) (AbD Serotec, Oxford, UK) and anti-beta Tubulin (1:1000 v/v) (Sigma Aldrich) for 2 h at room temperature, followed by incubation with a specific horseradish peroxidase (HRP)-conjugate secondary antibody (Dako, Golstrup, DK). The immune complexes were developed using enhanced chemiluminescence detection reagents (Amersham, Italy), according to the manufacturer's instructions and developed by an Image-Quant Apparatus (GE Healthcare). The protein bands were scanned and densitometrically analyzed with a GS-800 imaging densitometer (Bio-Rad Laboratories, CA, USA).

### Assessment of mechanical allodynia

Rats were individually placed on an elevated plastic mesh (1 cm^2 ^perforations) in a clear plastic cage and were allowed to adapt to the testing environment for at least 15 min. Von Frey hairs (Semmes-Weinstein monofilaments, 2 Biological Instruments, Italy) with calibrated bending forces (1.479, 2.041, 3.63, 5.495, 8.511, 11.749, 15.136 and 28.84 g) were used to deliver punctuate mechanical stimuli of varying intensity. The von Frey hairs were applied to the back of the rats, in the middle of and around the granulomatous tissue formed. Each stimulus was applied for approximately 1 s with an interstimulus interval of 5 s. Withdrawal responses evoked by each monofilament were obtained from five consecutive trials. Voluntary movement, associated with the locomotion, was not counted as a withdrawal response. Mechanical allodynia was defined as a significant decrease in the withdrawal threshold to the von Frey hair application. A 28.84-g hair sample was selected as the upper limit cut-off for testing. Mechanical allodynia was also measured by counting the frequency of withdrawals induced by 10 consecutive applications of the same Von Frey filament (26 g). Only robust and immediate withdrawal responses to the stimulus were considered as pain responses which were monitored for a period of 60 min by experimenters who were blinded to the treatment.

### ELISA essay

ELISA essay was performed on supernatants of centrifuged implanted sponges. Nunc Maxisorp 96-well microtiter plates (Gibco, Paisley, UK) were coated overnight at 4°C with 1 μg/well of specific monoclonal antibody anti-NGF, diluted in 0.5 M Na_2_CO_3_. The wells were washed three times and then blocked with phosphate-buffered saline (PBS) containing 0.05% (v/v) Tween 20 and 0.3% Fetal Bovine Serum (ELISA buffer) for 30 min at room temperature. The standards and samples in a volume of 100- μl were added then incubated at 37°C for 1.5 h. The standard curve was generated using 0.1 ng to 1 μg/well of purified protein, diluted in ELISA buffer. Following four washes with ELISA buffer, the monoclonal antibodies were diluted and added to the wells for 1 h. The wells were washed four more times and then incubated for 1 h with the secondary antibody: anti-mouse Ig, biotinylated species-specific F(ab8)2 fragment from donkey (Amersham, Milan, Italy), diluted 1:1000. Following a further four washes, the wells were incubated for 1 h with 100 μl streptavidinbiotinylated horseradish peroxidase complex (Amersham, Milan, Italy) diluted 1:1000 (v/v) in ELISA buffer. After a final six washes in ELISA buffer, 200 μl of a 0.4 mg/mL solution of *o*-phenylenediamine dihydrochloride (OPD, Sigma, Italy) in 0.05 M phosphate citrate buffer was added to each well and the colour was allowed to develop for up to 10 min. The colour reaction was stopped by addition of HCl and optical densities at 490 nm were measured using a microplate reader.

### Immunohistochemistry

Under pentobarbital anaesthesia, animals were transcardially perfused with saline solution followed by 4% paraformaldehyde in 0.1 M phosphate buffer. Dorsal root ganglia (DRGs) were excised, post- fixed for 4 h in the perfusion fixative, cryoprotected for 72 h in 20% sucrose in 0.1 M phosphate buffer and frozen in O.C.T. embedding compound. Transverse sections (12 μm) were cut using a cryostat and thaw-mounted onto glass slides. Slides were incubated overnight with primary antibody solutions for the pro-inflammatory markers: TNF-α (mouse anti-TNF-α 1:100 v/v, Sigma-Aldrich, Missouri, USA.); NGF (mouse anti-NGF 1:100 v/v, Oncogene Research Products, California, U.S.A) COX-2 (rabbit anti-COX2, Santa Cruz, California, USA) co-labeled with TRPV1 (goat anti-TRPV1, Santa Cruz, California, U.S.A); satellite cells (rabbit anti-glutamine synthetase, Abcam, Cambridge, UK). Following incubation, sections were washed and incubated for 3 h with secondary antibody solution (goat anti-rabbit, or donkey anti-rabbit or donkey anti-mouse, IgG-conjugated Alexa FluorTM 488 and 568; 1:1000; Molecular Probes, USA). Slides were washed, fitted with cover-slips with Vectashield mounting medium (Vector Laboratories, USA) and examined under a Leica fluorescence microscope. The double staining using secondary antibody from the same source was performed following the Tyramide Signal Amplification (TSA Plus Fluorescence System; PerkinElmer Life and Analytical Sciences, MA, USA) [24]. A negative control, performed using the secondary antibody alone, did not reveal any positive profile for all the markers analyzed.

### Statistical analysis

Results were expressed as the mean ± SEM of *n *animals where each value is the average of responses in duplicate sites. Statistical comparisons were made by T-Test or one way-ANOVA followed by Bonferroni's test for multiple comparisons and *p *< 0.05 was considered to be statistically significant. Pain responses to Von Frey hair stimuli between different treated groups of rats were performed using the Wilcoxon signed-ranks test and p < 0.05 was considered statistically significant.

In all set of experiments, analysis of linear regression was performed to evaluate a concentration-dependent relationship.

## Results

### Effect of PEA on λ-carrageenin-activated mast cells

**Figure 1:**
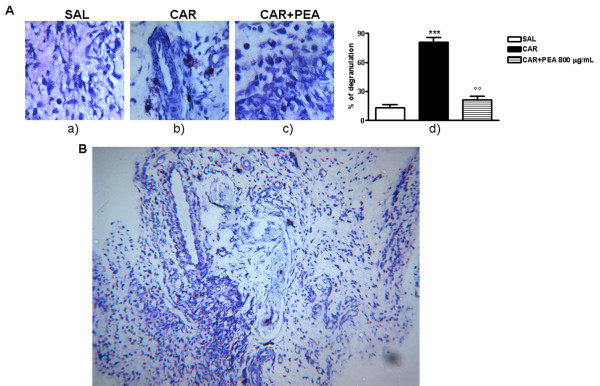
**Effect of PEA on λ-carrageenin-induced mast cell activation**. **"**A" shows the effect of PEA on λ-carrageenin-induced mast cell (MC) activation. MC degranulation was evaluated by microscopy. Connective MCs stained with 0.05% (w/v) toluidine blue and counterstained with 0.1% (w/v) nuclear fast red (magnification 100×). A differentiation between non degranulated (deep blue) and degranulated (light blue) MC was evaluable (a; b; c). In d) is shown the percentage of MC degranulation (ratio between degranulated and non degranulated MC). Results are expressed as mean ± SEM of 3 experiments. ***p < 0.001, °°p < 0.01. "B" shows high magnification of a representative histological analysis of granulomatous tissues stained with toluidine blue in which the presence of degranulated MCs is clearly visible close to blood vessels and nerves.

The histological analysis of λ-carrageenin-induced granuloma tissue, stained with toluidine blue, showed a massive degranulation of MCs (light blue stained cells, i.e. degranulated MCs, in comparison to the deep blue stained cells, i.e. non-degranulated MCs) compared with the saline controls. A significant inhibition of MC degranulation in granulomas of animals treated with PEA (800 μg/mL) was obtained (Figure 1A). At high magnification, the histological image of λ-carrageenin-induced granulomatous tissues showed that degranulated MCs were mainly localized not only in the proximity of the blood vessels but were also in close contact with nerves (Figure 1B).

### Effect of PEA on λ-carrageenin- induced NGF expression

**Figure 2:**
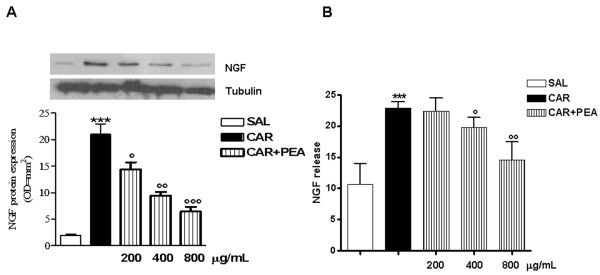
**Effect of PEA on λ-carrageenin-induced NGF expression and release in granulomatous tissue r 96 h after PEA administration**. (A) Representative Western blot analysis and relative densitometric analysis of NGF. Tubulin expression is shown as a control. (B) ELISA assay of NGF release in granulomatous tissue. Data are representative of 3 separate experiments. Results are expressed as mean ± SEM of 3 experiments.; *p < 0.05, **p < 0.01, ***p < 0.001 vs. saline.; °p < 0.05, °°p < 0.01, °°°p < 0.001 vs. λ-carrageenin alone.

NGF protein expression mainly released by activated MCs, evaluated by Western blot analysis, was increased in λ-carrageenin granulomatous tissues compared with saline controls; PEA treatment (200, 400, 800 μg/mL) produced a concentration-dependent significant reduction in NGF protein expression (Figure 2A). PEA treatment also significantly reduced, in a concentration-dependent manner, the release of NGF in λ-carrageenin-treated tissues, as measured by ELISA (Figure 2B).

### Effect of PEA on the formation of new nerve fibres

**Figure 3:**
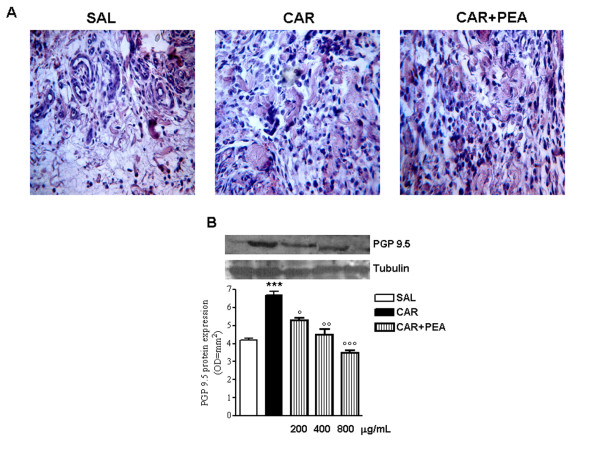
**Effect of PEA on λ-carrageenin-induced neurogenesis**. (A) Nerves formation was evaluated in representative histological analysis of granulomatous tissue stained with hematoxyl and eosin. Fields are representative of 3 separate experiments. Original magnification, 100×. (B) Representative Western blot analysis and relative densitometric analysis of PGP 9.5 protein expression, as a marker of neuronal cells. Results are expressed as mean ± SEM of 3 experiments. *p < 0.05, **p < 0.01, ***p < 0.001 vs. saline; °p < 0.05, °°p < 0.01, °°°p < 0.001 vs. λ-carrageenin alone.

Since the above findings showed the close proximity of MCs to nerves, we went on to study the effect of λ-carrageenin-induced MCs mediator release on new nerve fibres formation in granulomas. Histological analysis of granulomatous tissue stained with hematoxylin/eosin showed in the λ-carrageenin group an altered presence, shape and size of the nerve fibres, in comparison with saline controls that was restored by treatment with PEA (800 μg/mL) (Figure 3A).

The decrease in neurogenesis produced by PEA was also paralleled, thus confirmed, by a reduction in PGP 9.5 protein expression, a cytoplasmic protein present in neurons and neuroendocrine cells, which could be usedto visualize several different populations and subtypes of nerves. Western blot analysis revealed that PEA (200, 400, 800 μg/mL) acted in a concentration-dependent manner to reduce λ-carrageenin-induced PGP 9.5 expression (Figure 3B).

### Effect of PEA on mechanical allodynia

**Figure 4:**
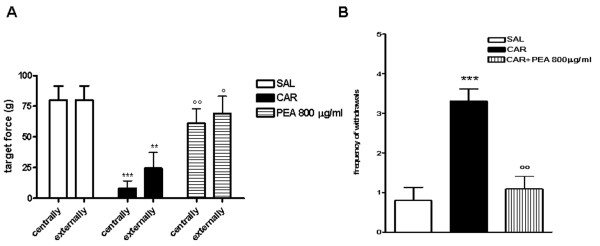
**Effect of PEA on λ-carrageenin-induced hyperalgesia**. Mechanical allodynia was evaluated by von Frey filament as (A) a target force both centrally and externally to granulomatous tissue and as (B) the frequency of withdrawals induced by 10 consecutive applications of the same von Frey filament. Results are expressed as mean ± SEM of 3 experiments. *p < 0.05, **p < 0.01, ***p < 0.001 vs. saline; °p < 0.05, °°p < 0.01, °°°p < 0.001 vs λ-carrageenin alone.

We then tested the pain response occurring in λ-carrageenin-induced granuloma with Von Frey filament measuring the pain response evoked by the application of different target forces, both centrally and externally to granulomas compared with saline-treated control rats. The treatment with PEA (800 μg/mL) markedly reduced pain responses both at the centre of and externally to granulomatous tissue, showing that this compound is able to control inflammatory pain (Figure 4A). The analgesic properties of PEA were then confirmed by its efficacy in reducing the frequency of withdrawals induced by 10 consecutive applications of the same target force Von Frey filament (Figure 4B).

### Effect of PEA on DRGs biomolecular and morphological changes

**Figure 5:**
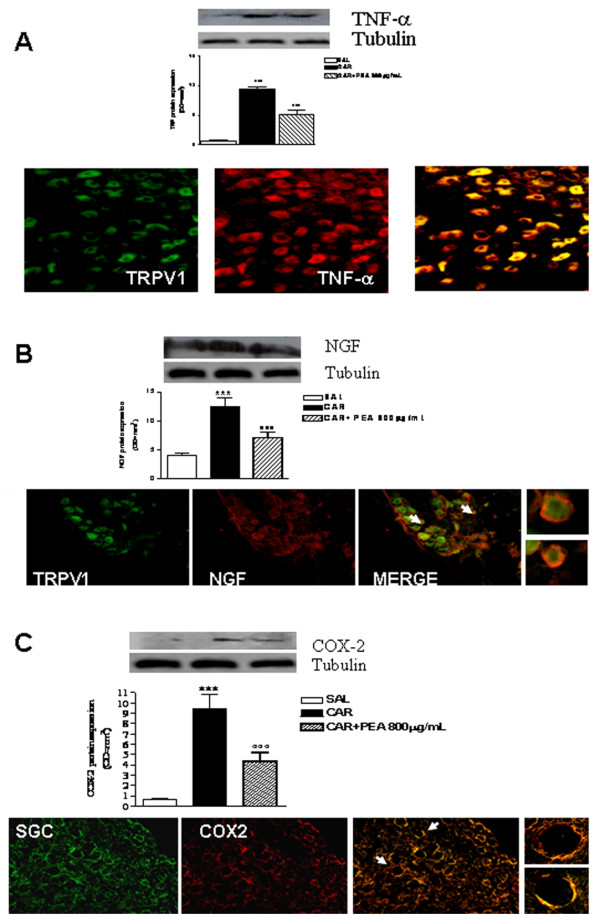
**Effect of PEA on DRG biomolecular and morphological changes induced by granuloma**. The effect of granulomatous inflammation on the biomolecular and morphological changes in the DRGs was associated with increased expression of TNF-α (A), NGF (B) and COX-2 (C). The levels of all proteins studied were significantly reduced by PEA treatment as revealed by Western blot analysis (A, B and C upper panels). Our results show that TNF-α and NGF were expressed by neurons as revealed by double labeling with TRPV1 receptor expressed in the DRG (A and B bottom panel) while COX-2 was highly expressed by satellite glial cells identified by glutamine synthetase staining (C bottom panel). Results are expressed as mean ± SEM of 3 experiments. ***p < 0.001 vs saline; °°°p < 0.001 vs λ-carrageenin alone.

We evaluated the effect of λ-carrageenin-induced granuloma on the biomolecular and morphological changes in the dorsal root ganglia (DRGs) directly receiving sensory input from *dermatomera *(skin segments) affected by granuloma formation. We found that granuloma-induced mechanical allodynia was associated with increased expression of the pro-inflammatory markers TNF-α, NGF and COX-2 in the DRGs. All these protein levels were significantly reduced by PEA treatment as revealed by Western blot analysis (Figure 5). We used an immunohistochemical approach in order to investigate the citotypes involved in the granuloma-induced DRGs sensitization. Our results showed that TNF-α and NGF were expressed by neurons as revealed by double labeling with *transient receptor potential vanilloid 1 *(TRPV1) expressed by nociceptive neurons in the DRG [25] (Figure 5A, B), while COX-2 was highly expressed by satellite glial cells as shown by glutamine synthetase staining (Figure 5C).

## Discussion

Based on our previous reports regarding the ability of PEA [22] and its analogue, Adelmidrol [23], to prevent granuloma formation by the control of MC activation in rats, the study described in the present paper was aimed to extend our knowledge about the pharmacological effects of PEA, focusing on mechanical allodynia that may occur during chronic inflammatory conditions [22,23]. The histological analysis of granulomatous tissue highlighted a massive presence of degranulated MCs, not only closely associated with blood vessels, as previously described by us [23], but also in tight contact with nerve fibres. This last observation was in accordance with another study hypothesizing that there is a close relationship between MCs and nerve fibres occurs in modulation of neuronal plasticity [2]. Accordingly, we also investigated the effect of PEA, based on the well described control of MC activation, on nerve fibre activity in granuloma. Similarly to Adelmidrol, indeed, PEA administration reduced MC activation and the subsequent release of bioactive mediators within and around granulomatous tissue [1]. Particular emphasis, in this paper, was given to NGF derived from MCs, which is considered one of the key mediators of neuronal plasticity and nerve fibre sensitization [26,27]. The role exerted by NGF in pain signaling is now well understood: low doses of NGF produce pain and hyperalgesia in adult animals; in rodents, thermal and mechanical hyperalgesia develops after systemic NGF administration [28]; moreover, NGF produces sensitization of nociceptors both directly (after activation of its specific receptors on nociceptive fibres) and indirectly, through other peripheral cell types. The direct mechanism involves both altered gene expression and post-translational regulation of receptors, including TRPV1 [29]. Moreover, NGF can trigger a number of peripheral immune cell types expressing trkA, including MCs. In fact, NGF can produce degranulation and increase proliferation of MCs [30], resulting in an autocrine/paracrine loop that sustains inflammation and hyperalgesia [31]. Our data showed that PEA, by modulating MC degranulation, reduced, in a concentration-dependent manner, NGF protein expression and release in the chronically inflamed tissues. The reduction in NGF exerted by PEA in our model can be explained with different mechanisms of action, an ALIA mechanism played on MCs present in granulomatous tissues in accordance with the original hypothesis proposed by Facci et al. [19], or by the "entourage effect" operated by PEA on other endocannabinoid receptor targets, i.e. CB_1 _or TRPV1 receptors as previously demonstrated in a model of neuropathic pain by Costa et al. [32] or, finally, by the binding to an orphan receptor also expressed on mast cells, the G-protein coupled receptor-55 (GPR-55) [33].

The effect of PEA on NGF release leads to a reduction in the number of nerve fibres in λ-carrageenin-induced granulomatous tissue. These results are consistent with the well known role of NGF, able to produce nerve sprouting within and close to injured tissue [34], an effect due to the neurotrophic ability of NGF to affect neuronal growth, neuronal survival and axonal outgrowth [8]. The decrease in the number of nerve fibres observed in the histological analysis of PEA-treated animals was also seen to occur in parallel with a concentration-dependent decrease in PGP 9.5 protein expression, a cytoplasmic protein present in neurons and neuroendocrine cells, useful to visualize several different populations and subtypes of nerves, which confirmed our previously discussed results.

Therefore, it seems possible to justify the lower pain sensitivity exhibited by rats treated with PEA considering the reduced number of nerve fibres obtained with PEA treatment which is associated with a decrease in NGF. In fact, NGF is considered to be not only a promoter of neuronal sprouting, but it is also involved in neural sensitization since it increases the excitability and the firing of sensory neurons [35-37]. Our results are in agreement with previous data showing that administration of PEA produces antinociception in the formalin test [38], reduces inflammatory hyperalgesia and oedema by inhibiting MC degranulation [39] and inhibits mechanical hyperalgesia after intraplantar carrageenin challenge in animals [40]. Moreover, the results reported in the present study are consistent with a recent study demonstrating that the anti-hyperalgesic action of PEA in a model of neuropathic pain depends, at least in part, on the reduction in NGF up-regulation [32].

Growing evidences indicate the role of glial and microglial cells in the induction and maintenance of pathological pain-associated allodynia. Also at DRG level, the satellite glial cells, which in fact represent the peripheral glial cells (astrocytes), seem to play a key role in the maintenance of neuropathic pain-associated mechanical allodynia [41].

Further insight can be obtained into PEA analgesic effects since it was also able to prevent DRG neural sensitization, here indirectly suggested by immunohistochemistry and western blotting for TNF-α and NGF, as well as the COX-2 in satellite cells. In fact, a significant increase in TNF-α, NGF and COX-2 was found in DRGs isolated in granuloma from rats treated with λ-carrageenin, compared with control animals. Interestingly, double staining images showed that TNF-α and NGF have TPRV1-positive profiles, corresponding to neurons [25], while COX-2 protein was expressed by satellite glial cells in DRGs. These results underline the fact that the induction of granulomatous tissue triggers significant functional changes in DRG neurons which is also corroborated by the involvement of satellite cells in releasing pro-inflammatory molecules. In actual fact, NGF itself has been shown to sensitize dorsal horn neurons after intramuscular injection [42]. PEA, by switching off the inflammation in λ-carrageenin-induced granuloma formation, might be responsible for the inhibition of DRG neuronal sensitization, as suggested by the reduced expression of NGF, COX-2 and TNF-α, as well as by the decreased mechanical allodynia in animals treated with PEA. These observations are consistent with a previous study demonstrating that intraplantar administration of PEA preferentially suppresses spinal neuronal sensitization evoked by hindpaw formalin administration [43].

Hence, the present data support an analgesic role played by PEA in several models of pain, like persistent somatic inflammatory pain, visceral inflammatory pain and neuropathic pain, and recognize, for the first time, in MCs the leading cell-type affected by PEA in this model of chronic inflammation-dependent pain [44]. Thus, according to our results it is reasonable to propose the use of PEA and its congeners in the treatment of several painful conditions, especially those involving MC degranulation and NGF release.

## Competing interests

This work was partially supported by the Epitech Group.

## Authors' contributions

DDF designed *in vivo *experiments and performed behavioural test. LL designed and performed immunohistochemistry analysis. MC performed biochemical analysis. EP performed the isolation of DRGs. MPC performed and critically discussed all histological analysis. VDN participated in the design of the study and in the revision of the text. SM critically contributed to all the aspect of the MS concerning allodynia and pain. TI revised all manuscript performing the final critical analysis to the text. All authors read and approved the final manuscript
